# Presence of Armed School Officials and Fatal and Nonfatal Gunshot Injuries During Mass School Shootings, United States, 1980-2019

**DOI:** 10.1001/jamanetworkopen.2020.37394

**Published:** 2021-02-16

**Authors:** Jillian Peterson, James Densley, Gina Erickson

**Affiliations:** 1 Department of Criminal Justice, Hamline University, St Paul, Minnesota; 2 School of Law Enforcement and Criminal Justice, Metropolitan State University, St Paul, Minnesota

## Abstract

This cross-sectional study examines whether there is an association between having an armed guard at school and the prevalence of deaths and injuries during school shootings and attempted shootings.

## Introduction

After deadly school shootings at Columbine, Sandy Hook, and Parkland, many states mandated School Resource Officers or provided funding for districts to hire them.^1^ Lawmakers also considered arming teachers. Florida now requires a law enforcement officer or trained school guardian in every school.^2^

By examining every recorded incident where one or more people was intentionally shot in a school building during the school day, or where a perpetrator came to school heavily armed with the intent of firing indiscriminately, we examine the association between the presence of an armed officer on scene and the severity of shootings in K-12 (kindergarten through 12th grade) schools.

## Methods

This cross-sectional study was deemed exempt by the Hamline University institutional review board and granted a waiver of informed consent because it only used publicly available records for coding. This study followed the Strengthening the Reporting of Observational Studies in Epidemiology (STROBE) reporting guideline.

We examined each identified case where at least one person was intentionally shot in a school building during a school day or a person arrived at school with the intent of firing indiscriminately (133 total cases) from 1980 to 2019 as reported by the public K-12 School Shooting Database.^3^ We focused on offender motive, an armed guard on scene during the shooting, the number and type of firearms the perpetrator used, and other factors. Following prior work on public mass shootings,^4^ the codebook was piloted on a random sample of cases. Each shooting was investigated twice by separate coders working independently. Data were merged and differences were resolved via consensus. The cases were then divided, independently checked, and sources triangulated.

Negative binomial regression models predicting number injured and killed were used to account for the overdispersion of count data; missing data (<12% on any variable) are reported in Table 1 and imputed in multivariate models using multiple imputation in Stata software version 16 (StataCorp).^5^ All tests indicate significance at the *P* < .05 level. All tests of significance are model parameters in Table 2. Data analyses were performed from November to December 2020.

**Table 1.** Descriptive and Missing Case Information for All Variables

Variable	Cases, No. (%)	Persons injured, mean (SD), No.	Persons killed, mean (SD), No.	Missing cases, No. (% imputed)
Persons killed per case, mean (SD) [range], No.	NA	NA	1.34 (3.25) [0-27]	NA
Persons injured per case, mean (SD) [range], No.	NA	3.15 (5.06) [0-32]	NA	NA
Weapons per case, mean (SD) [range], No.	1.63 (1.22) [1-8]	NA	NA	3 (2.24)
Armed officer	29 (23.58)	3.86 (5.45)	2.07 (4.16)	11 (8.21)
Lockdown drills	53 (44.92)	2.91 (4.75)	1.77 (4.63)	16 (11.94)
Targeted	57 (47.11)	2.74 (3.54)	1.11 (1.73)	13 (9.70)
No. of shooters				
One	124 (92.54)	2.94 (4.93)	1.29 (3.18)	NA
More than one	10 (7.46)	5.80 (6.12)	2.00 (4.16)	NA
Known weapon type				
Any AR or SMG	14 (10.45)	7.79 (9.69)	5.36 (8.05)	NA
Any handgun	92 (68.66)	3.18 (3.18)	1.45 (3.24)	NA
Any shotgun	29 (21.64)	3.72 (5.20)	1.79 (3.37)	NA
Any rifle	23 (17.16)	3.74 (5.56)	0.87 (1.49)	NA
Region				
South	39 (29.10)	3.36 (4.45)	1.41 (3.17)	NA
Midwest	35 (26.12)	1.60 (1.85)	0.83 (1.69)	NA
Northeast	19 (14.18)	1.37 (1.54)	2.05 (6.16)	NA
West	41 (30.60)	5.10 (7.41)	1.39 (2.38)	NA
Urbanicity				
Urban	34 (25.37)	2.82 (4.46)	0.56 (1.05)	NA
Suburban	58 (43.28)	3.60 (5.73)	1.67 (4.46)	NA
Rural	42 (31.34)	2.79 (4.57)	1.52 (2.24)	NA
School type				
Elementary	17 (12.69)	5.53 (8.44)	2.94 (6.44)	NA
High school	81 (60.45)	3.15 (4.89)	1.21 (2.67)	NA
Middle or combined	36 (26.87)	2.03 (2.86)	0.89 (1.86)	NA
Institution type				
Public	122 (91.04)	3.28 (5.24)	1.42 (3.37)	NA
Private/other	12 (8.96)	1.83 (2.44)	0.58 (1.44)	NA

Abbreviations: AR, assault rifle; NA, not applicable; SMG, submachine gun.

**Table 2.** Negative Binomial Regression Results for Number Injured and Number Killed in School Mass Shootings

Variable	Injured		Killed	
	IRR (95% CI)	*P* value	IRR (95% CI)	*P* value
Lockdown drills	0.90 (0.55-1.49)	.69	0.70 (0.35-1.40)	.32
Armed officer	1.21 (0.69-2.11)	.51	2.96 (1.43-6.13)	.003
No. of weapons	1.22 (0.97-1.54)	.09	1.34 (1.00-1.79)	.048
Targeted	0.94 (0.58-1.52)	.79	0.91 (0.48-1.73)	.77
More than one shooter	1.62 (0.73-3.59)	.24	1.008 (0.32-3.14)	.99
Weapon type				
Any AR or SMG	2.27 (1.07-4.81)	.03	12.84 (4.88-33.74)	<.001
Any handgun	1.37 (0.73-2.57)	.33	4.85 (2.02-11.63)	<.001
Any shotgun	1.29 (0.66-2.50)	.45	1.448 (0.61-3.41)	.40
Any rifle	1.39 (0.71-2.72)	.34	1.497 (0.58-3.87)	.41
Region				
South	1.84 (0.96-3.52)	.07	1.076 (0.47-2.49)	.86
Northeast	0.80 (0.35-1.81)	.59	1.186 (0.46-3.09)	.73
West	2.20 (1.22-3.96)	.009	0.907 (0.43-1.93)	.80
Urbanicity				
Urban	1.14 (0.64-2.03)	.65	0.628 (0.27-1.46)	.28
Rural	0.99 (0.56-1.75)	.97	2.303 (1.08-4.91)	.03
School type				
Elementary	1.39 (0.70-2.74)	.34	1.328 (0.58-3.05)	.50
Middle/combined	0.76 (0.43-1.34)	.34	0.898 (0.41-1.96)	.79
Institution type				
Private/other	0.48 (0.19-1.20)	.12	0.192 (0.04-0.91)	.04

Abbreviations: AR, assault rifle; IRR, incidence rate ratio; SMG, submachine gun.

## Results

This study examined a total of 133 cases of school shootings and attempted school shootings from 1980 to 2019. Perpetrators’ ages ranged from 10 to 53; however, only 16 shooters (11%) were aged 22 years or older. Ninety-four perpetrators (70%) were current students, and 21 perpetrators (15%) were former students. Of all perpetrators, 83 (76%) were White and 148 (98%) were male. Of 121 cases with full information, 57 (47.11%) were targeted shootings. There were 134 shootings, 12 with more than one shooter. A mean (SD) of 1.34 (3.25) people per case were killed and 3.15 (5.06) per case were injured, with a mean (SD) of 1.63 (1.22) weapons per shooting (primarily handguns; 68.66% [92 of 134]). An armed guard was on scene in 23.58% of shootings (29 of 123) (Table 1).

Based on theory, multivariate models include the presence of an armed guard and control for region, school type (public, nonpublic), and grade level (high school, elementary, other); location (urban, suburban, rural); use of lockdown drills; if the attack was targeted; total number of weapons brought to the scene; number of shooters; and weapon type. Results are presented as incident rate ratios in Table 2 and show armed guards were not associated with significant reduction in rates of injuries; in fact, controlling for the aforementioned factors of location and school characteristics, the rate of deaths was 2.83 times greater in schools with an armed guard present (incidence rate ratio, 2.96; 95% CI = 1.43-6.13; *P* = .003).

## Discussion

This study had some limitations. It is limited by its reliance on public data, lack of data on community characteristics, and inability to measure deterred shootings (nonevents). However, the data suggest no association between having an armed officer and deterrence of violence in these cases. An armed officer on the scene was the number one factor associated with increased casualties after the perpetrators’ use of assault rifles or submachine guns.

The well-documented weapons effect explains that the presence of a weapon increases aggression.^6^ Whenever firearms are present, there is room for error, and even highly trained officers get split-second decisions wrong. Prior research suggests that many school shooters are actively suicidal, intending to die in the act, so an armed officer may be an incentive rather than a deterrent.^4^ The majority of shooters who target schools are students of the school, calling into question the effectiveness of hardened security and active shooter drills. Instead, schools must invest in resources to prevent shootings before they occur.
