# Error in Methods

**DOI:** 10.1001/jamanetworkopen.2022.12528

**Published:** 2022-04-25

In the Research Letter titled “Presence of Armed School Officials and Fatal and Nonfatal Gunshot Injuries During Mass School Shootings, United States, 1980-2019,”^1^ published February 16, 2021, there was an error in the wording used to describe the study sample in the Methods section. The correct description of the sample is, “We examined each identified case where more than one person was intentionally shot in a school building during a school day or a person arrived at school with the intent of firing indiscriminately (133 total cases) from 1980 to 2019 as reported by the public K-12 School Shooting Database.” This article has been corrected.^1^
